# Viability of dietary substitution of live microalgae with dry *Ulva rigida* in broodstock conditioning of the Pacific oyster (*Crassostrea gigas*)

**DOI:** 10.1242/bio.035923

**Published:** 2018-08-20

**Authors:** Ana Rato, Sandra Joaquim, Tânia G. Tavares, Zita E. Martins, A. Catarina Guedes, Luís F. Pereira, Jorge Machado, A. Margarete Matias, José F. M. Gonçalves, Paulo Vaz-Pires, Leonardo J. Magnoni, Rodrigo O. A. Ozório, Domitília Matias

**Affiliations:** 1 Interdisciplinary Centre of Marine Environmental Research (CIIMAR), University of Porto, Terminal de Cruzeiros do Porto de Leixões, Av. General Norton de Matos s/n, 4450-208 Matosinhos, Portugal; 2 Department of Sea and Marine Resources, Portuguese Institute for Sea and Atmosphere (IPMA, I.P.), Av. 5 de Outubro s/n, 8700-305, Olhão, Portugal; 3 Laboratory of Engineering of Processes, Environment, Biotechnology and Energy (LEPABE), Rua Dr Roberto Frias, P-4200-264 Porto, Portugal; 4 Department of Chemical Sciences (LAQV/REQUIMTE), Faculty of Pharmacy, University of Porto, Rua de Jorge Viterbo Ferreira, 228, P-4050-313 Porto, Portugal; 5 Department of Aquatic Production, Abel Salazar Biomedical Sciences Institute (ICBAS), University of Porto, Rua Jorge de Viterbo Ferreira 228 4050-313 Porto, Portugal

**Keywords:** Pacific oyster, Conditioning, Dietary introduction, Dry macroalgae, Biochemical composition, Spawning

## Abstract

The current study evaluated the microalgae replacement by dry macroalgae (*Ulva rigida*) in the reproductive success and biochemical composition of the Pacific oyster (*Crassostrea gigas*) during broodstock conditioning. Five nutritional regimes were tested: 100% macroalgae (diet 1), 50% macroalgae+50% microalgae (diet 2), 25% macroalgae+75% microalgae (diet 3) and 100% microalgae (diet 4). An unfed group was used as a negative control. The microalgae blend was composed of 33% *Isochrysis galbana* and 67% diatoms (75% *Skeletonema costatum*+25% *Chaetoceros calcitrans*). Gonadal maturation was reflected in the physiological condition of the individuals. All treatments, except diet 1, showed an increase in condition index and were fully matured at the end of the trial, with the best physiological condition observed in oysters fed diet 3 and diet 4. Protein and total lipid content increased during the conditioning period, whereas glycogen content decreased. Oysters conditioned with diet 3 had higher protein and total lipid content and lower glycogen content than the other treatments. In addition, diet 3 showed the highest percentage of viable veliger larvae. The current study demonstrated that it is possible to replace 25% of microalgae with macroalgae in the broodstock conditioning, minimizing the operative cost in bivalve hatcheries.

This article has an associated First Person interview with the first author of the paper.

## Abstract (precis)

**Summary:** The use of an alternative diet with 25% microalgae replacement by dry macroalgae is beneficial during broodstock conditioning and allows the operation costs of bivalve hatcheries to be minimized.

## INTRODUCTION

Pacific oysters *Crassostrea gigas* (Thunberg, 1793), a native species from northeast Asia, was introduced worldwide, mainly to prevent a crisis resulting from the massive decline of indigenous populations and to sustain aquaculture industries (Boudry et al., 1998). Due to its biological characteristics, such as fast growth, high tolerance and ability to adapt to a wide range of environmental conditions, the Pacific oyster has become a high-value species in aquaculture worldwide (Fabioux et al., 2005; Kheder et al., 2010; Pogoda et al., 2013).

The zootechnical development for seed production in hatcheries is extremely important (Marshall et al., 2010) to provide juveniles of a high quality to bivalve producers. Hatchery production generally comprises three distinct phases: a broodstock conditioning period, which provides larvae for culturing, and a subsequent post-larval rearing phase (Fabioux et al., 2005; González-Araya et al., 2012; Helm et al., 2004; Marshall et al., 2010).

Broodstock conditioning, an essential step in hatchery procedure, aims to maximize the fecundity of breeding animals, while maintaining egg quality and consequent viability of the larvae (González-Araya et al., 2012; Utting and Millican, 1997).

The success of bivalve production in hatcheries is undeniably related to the quality and the quantity of food available (Delaporte et al., 2006; Helm et al., 2004). Energy reserves are of considerable importance in reproduction, and energy storage and utilization in bivalves are closely correlated to the quality of diet provided to adults, which consequently affects gonadal development, oocyte quality and larval viability (Anjos et al., 2017; Utting and Millican, 1997).

Several nutritional studies in many bivalve species (Anjos et al., 2017; González-Araya et al., 2011; González-Araya et al., 2012; Pronker et al., 2008; Utting and Millican, 1997) have focused in achieving the optimal algal composition to feed the broodstock, in order to accomplish optimum reproductive outcomes. The nutritional profile of the diet influences the physiology of bivalves, particularly the specific form of proteins, carbohydrates and especially lipids (Joaquim et al., 2011; Matias et al., 2009). Lipids are usually used as an energy source during gametogenesis (Delgado et al., 2004) and constitute the principal nutritional reserves in eggs and larvae (Helm et al., 1973; Matias et al., 2011).

It is common in hatcheries to feed bivalves with microalgae blends. Since microalgae species vary substantially in their nutritional value, the use of a cocktail of several species may enable us to create a nutritionally balanced feed (Brown and Robert, 2002; Knauer and Southgate, 1999; Spolaore et al., 2006).

Aquaculture of bivalves is strongly dependent on the production of live microalgae, which represents 30–40% of the operation costs (Coutteau and Sorgeloos, 1992), constituting an economic limitation due to high costs of production, culture instability and batch variability (Arney et al., 2015; Borowitzka, 1997; Guedes and Malcata, 2012). To overcome this constraint and to reduce the use of live microalgae in bivalve hatcheries, several research lines have focused on evaluating alternative diets (Arney et al., 2015; Boeing, 1997; Knauer and Southgate, 1999; Langdon and Önal, 1999; Parwadani-Aji, 2011). However, no commercial formulated diet for broodstock bivalves is currently available (Brown, 2002; Muller-Feuga, 2000; Pronker et al., 2008).

Macroalgae are considered as a food source both for human and animal nutrition, mainly due to their high nutritional value (Fleurence, 1999; Peng et al., 2015). Macroalgae nutritional values differ considerably with the species: red macroalgae contain a high level of proteins (35–47% of dry weight), followed by green macroalgae (10% and 25% of dry weight), and brown macroalgae (5–12% of dry weight) (Fleurence et al., 2012). In addition to protein content, some green macroalgae such as *Ulva* have high levels of mineral elements (calcium and magnesium) with nutritional value (Fleurence et al., 2012).

*Ulva* species have become important macroalgae, due to their nutritional properties (protein, minerals and vitamins) (Ortiz et al., 2006) and for their role in stress response and disease resistance (Fleurence et al., 2012). The introduction of *Ulva* as a dietary ingredient has being investigated for several fish species (Abdel-Warith et al., 2016; Ergün et al., 2009; Valente et al., 2006) and commercial marine invertebrates that feed on macroalgae, such as sea urchins (Cook and Kelly, 2007) and abalone (Bautista-Teruel et al., 2001; Bilbao et al., 2012; Kemp et al., 2015).

To evaluate the effect of substitution of live microalgae on broodstock conditioning of *C. gigas*, the present study replaced the live microalgae with commercial dry macroalgae *U. rigida* at various substitution levels (0, 25, 50 and 100%)*.* A microalgae blend consisting of diatoms (*Skeletonema costatum* and *Chaetoceros calcitrans*) and a flagellate *Isochrysis galbana* clone T.ISO was used as a positive control. This diet was formulated based in Anjos et al. (2017), where a diet predominantly consisting of diatoms elicited the best results in broodstock conditioning of *C. angulata*. Reproductive output of *C. gigas* and the biochemical composition (proteins, glycogen and total lipids) of diets and of oysters during conditioning were evaluated. The aim is to find an alternative broodstock conditioning diet that maximizes fecundity and oocyte quality, being suitable to be used in commercial hatcheries.

## RESULTS

### Diet composition

The nutritional composition of the diets (protein, total lipids and total carbohydrates) are presented in Table 1. Carbohydrates were the predominant constituent of all diets, followed by proteins and lipids. Results showed significant differences between the four experimental diets in all the parameters analyzed.

**Table 1.**
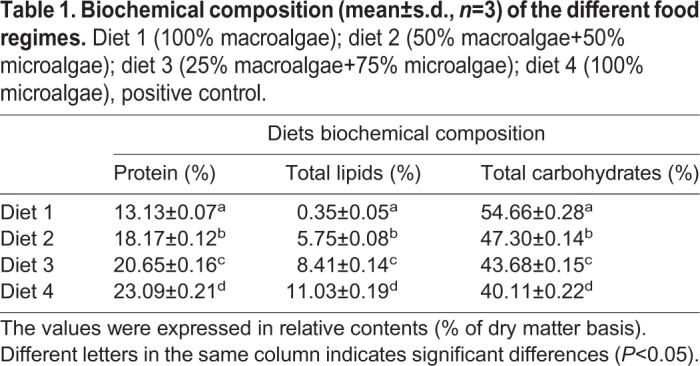
**Biochemical composition (mean±s.d., *n*=3) of the different food regimes.** Diet 1 (100% macroalgae); diet 2 (50% macroalgae+50%microalgae); diet 3 (25% macroalgae+75% microalgae); diet 4 (100% microalgae), positive control.

In general, the percentages of proteins and total lipids decrease with increasing inclusion of macroalgae in the diet (protein, ANOVA, *F*=2422.549, *d.f*.=3, *P*<0.05; total lipids, ANOVA, *F*=3940.955, *d.f*.=3, *P*<0.05). While the total carbohydrates presented a decrease with the increase of the inclusion of microalgae in the diet (ANOVA, *F*=22,736.202, *d.f*.=3, *P*<0.05).

### Broodstock gonadal development

**Fig. 1.**
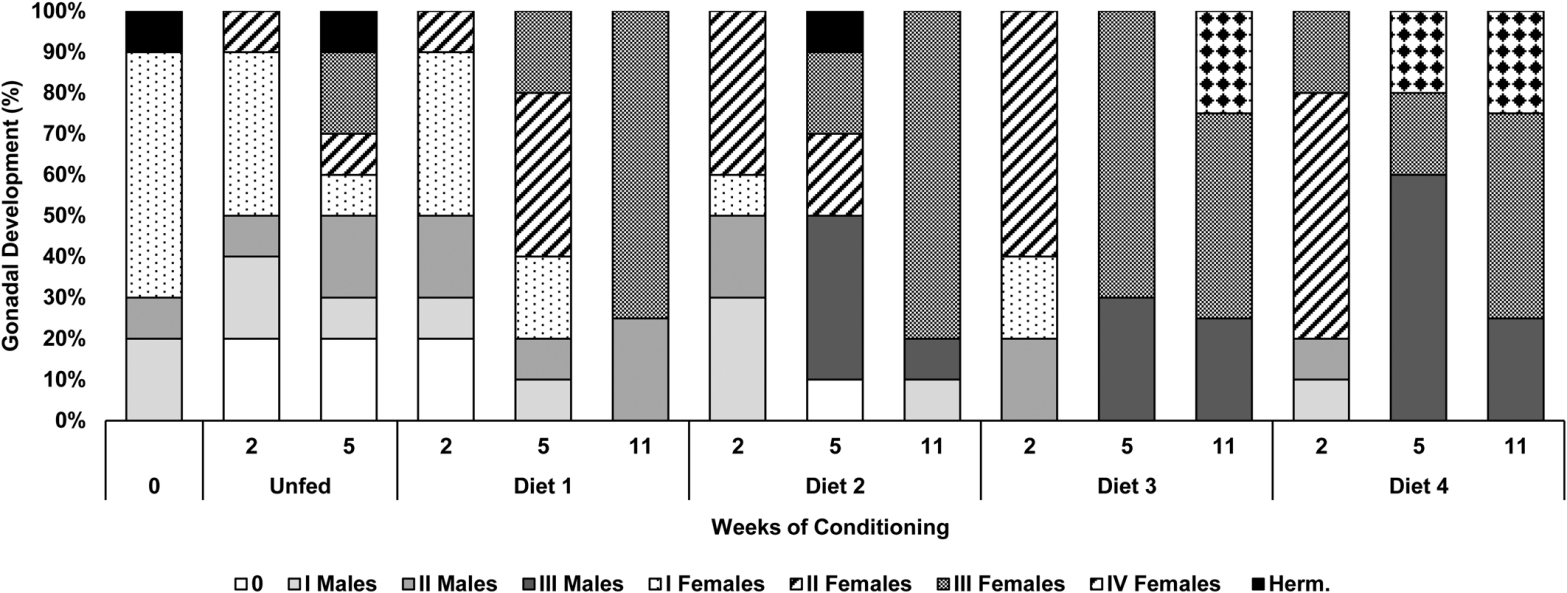
**Gonadal development (%) in *C. gigas* broodstock conditioned with different diets.** Unfed, 100% macroalgae (diet 1); 50% macroalgae+50% microalgae (diet 2); 25% macroalgae+75% microalgae (diet 3) and 100% microalgae (diet 4). Stage 0- resting stage; stage I- early gametogenesis; stage II- late gametogenesis; stage III- maturation; stage IV- spawning and reabsorbing; herm., hermaphrodite.

In general, there was a development in gonadal maturation observed in all treatments during the conditioning period (Fig. 1), with a higher homogeneity in late samplings, especially for oysters conditioned with diet 3 and 4.

When the conditioning started (week 0), 20% of males were in early gametogenesis (stage I) and 10% in late gametogenesis (stage II); 60% of individuals were females in early gametogenesis and 10% of the oysters exhibited male and female gametes, hence were considered as hermaphrodites.

In unfed group, there was a slight regression in gonadal development from week 0–2, with 20% of oysters at resting stage (stage 0). However, females showed slow gonadal development, with 10% of females at stage II and 40% at stage I, while males remained in the same gonadal stages. At week 5, it was observed that there was a development in gonadal maturation, with high intra-heterogeneity in maturation stages with 20% at resting stage, males 10% at early gametogenesis and 20% at late gametogenesis, females 10% at early gametogenesis, 10% at late gametogenesis and 20% at maturation (stage III). It was also observed in 10% of hermaphrodite individuals.

Oysters fed with 100% macroalgae (diet 1) exhibited, at week 2, a gonadal development similar to the unfed group. Nevertheless, the 100% macroalgae group showed a higher percentage of males at stage II (20%), whereas there were 10% in stage I. At week 5 20% of females were at early gametogenesis, 20% at late gametogenesis and 40% were mature females, while males were at early gametogenesis (10%) and late gametogenesis (10%). At week 11 an increase in gonadal development as well as a homogeneity in maturation stages were observed, where all females (75%) were mature (stage III) while all males (25%) were at stage II.

At week 2, from all oysters fed with 50% macroalgae and 50% microalgae (diet 2), 40% of females were at stage II, while 10% were at stage I. The same pattern was observed in males, i.e. 30% were at stage I and 20% at stage II. At week 5 there was a slight regression with 10% of individuals at resting stage. Nevertheless, all males (40%) were at stage III while females were at stages II and III (20% in each), with 10% of hermaphrodites. At week 11, all females were mature (stage III), whereas 10% of males were at stage I and 10% at stage III.

Oysters fed diet 3 and diet 4 showed a faster maturation in two weeks of conditioning than the other treatments. Males fed diet 3 were all at stage II (20%), whereas 10% of males from diet 4 were at stage I and 10% at stage II. Females from diet 3 group, were at stage I (20%) and stage II (60%), whereas in diet 4 group, 60% of females were in late gametogenesis and 20% were mature females (stage III). At week 5, all animals fed with diet 3 were mature at stage III. A higher percentage of mature males (stage III) was observed in diet 4 group (60%), while 20% females were in the maturation stage and 20% were in the spawning and reabsorbing stage (stage IV). At the end of the conditioning period, both diet 3 and diet 4 groups were composed of 25% of mature males (stage III) and females, 50% were at stage III and 25% stage IV.

### Condition index

**Fig. 2.**
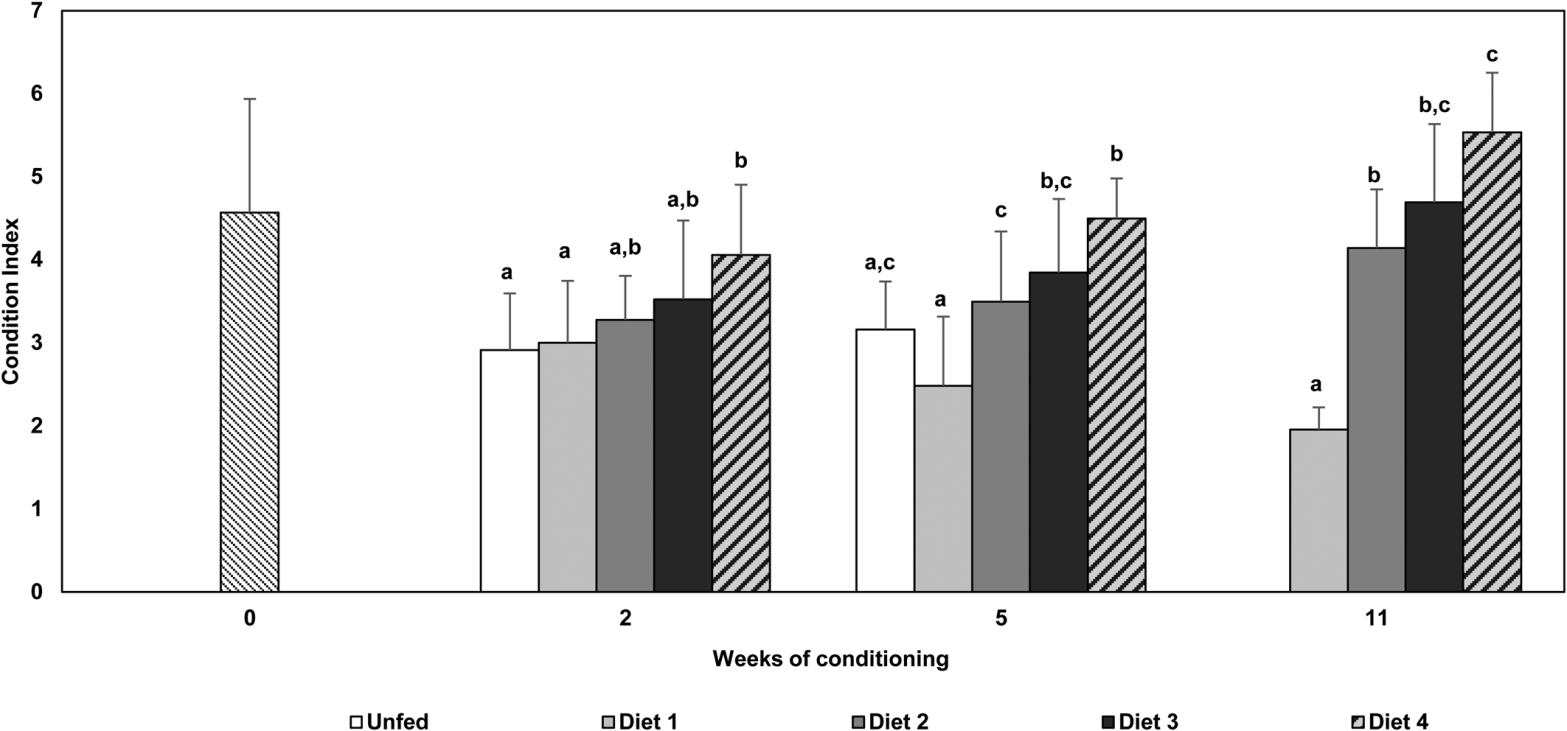
**Condition Index (mean±s.d.) in *C. gigas* broodstock conditioned with different nutritional regimes.** Unfed, 100% macroalgae (diet 1); 50% macroalgae+50% microalgae (diet 2); 25% macroalgae+75% microalgae (diet 3) and 100% microalgae (diet 4). Groups with different letters indicate significant differences (*P<*0.05).

The condition index of oysters from all treatments (Fig. 2) decreased from initial sampling (T0=4.57±1.37) to week 2 (unfed–2.91±0.68; diet 1–3.00±0.75; diet 2–3.27±0.53; diet 3–3.52±0.95; diet 4–4.06±0.85), being less pronounced in oysters fed with 100% microalgae (diet 4). Significant differences were observed between diet 4 and unfed group and 100% macroalgae (K–W, H=11.502, *d.f.*=4*; P*=0.021). Condition index of oysters fed with diet 1 showed a continuous decrease until the end of the conditioning period (T5: 2.47±0.83; T11: 1.96±0.27). At week 5 and 11, significant differences between diet 1 and diets 2, 3 and 4 were observed (week 5: ANOVA, *F*=9.503, *d.f*.=4, *P*<0.001; week 11: ANOVA, *F*=23.693, *d.f*.=3, *P*<0.001). At week 5, condition index of the other dietary treatments showed a general increase (unfed–3.16±0.58; diet 2–3.49±0.85; diet 3–3.85±0.89; diet 4–4.50±0.48). This pattern was also observed also at week 11. At weeks 5 and 11, diet 4 showed the highest value of condition index, followed by diet 3 (T11: diet 2–4.14±0.70; diet 3–4.69±0.94; diet 4–5.53±0.72). At week 5 and 11 significant differences were observed between the diet 4 group and the other dietary groups, with the exception of diet 3 group at week 11.

### Broodstock biochemical composition

The analysis of biochemical composition of broodstock revealed a highly heterogeneous response to the different diets, where proteins were the predominant compound of the individuals, followed by total lipids and then by glycogen (Table 2).

**Table 2.**
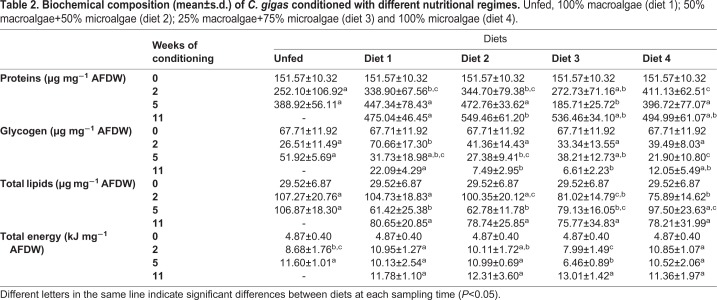
**Biochemical composition (mean±s.d.) of *C. gigas* conditioned with different nutritional regimes.** Unfed, 100% macroalgae (diet 1); 50% macroalgae+50% microalgae (diet 2); 25% macroalgae+75% microalgae (diet 3) and 100% microalgae (diet 4).

Protein content ranged from 151.57±10.32 µg mg^−1^ AFDW to 549.46±61.20 µg mg^−1^ AFDW. In general, an increase in protein content in all dietary groups was observed. From week 2, the most pronounced increase in protein content was observed in diet 4 group, which was significantly different from the other dietary treatments (ANOVA, *F*=10.885, *d.f.*=4, *P*<0.001), whereas a less pronounced increase was observed in diet 3 group. The unfed group were significantly different from the diet 1 and diet 2 groups (ANOVA, *F*=10.885, *d.f*.=4, *P*<0.001). At week 5 (T5), oysters fed with diet 3 and 4 exhibited a decrease in protein content, with the diet 3 group exhibiting significant differences from the remaining dietary groups (K–W, H=47.050, *d.f*.=4, *P*<0.001). Both groups have recovered at the end of the conditioning period (T11), with diet 3 group exhibiting the most remarkable increase. The remaining treatments showed an increase in all sampling times.

Glycogen content showed an opposite pattern of that observed in proteins content, in general, glycogen content decreased during the conditioning period. Glycogen varied between 67.71±11.92 µm mg^−1^ AFDW at the beginning of the trial and 6.61±2.23 µm mg^−1^ AFDW at the end. After two weeks of conditioning, there was a decrease in glycogen content in all dietary groups, with exception of diet 1 group, which revealed a significant increase when compared with the other treatments (K-W, H=42.526, *d.f*.=4, *P*<0.001). At week 5 (T5), unfed and diet 3 groups showed an increase in glycogen content, while the other treatments decreased. Unfed group was significantly different from diet 2 and diet 4 (K-W, H=24.929, *d.f.*=4, *P*<0.001), whereas diet 3 and diet 4 were different from each other (K-W, H=24.929, *d.f.*=4, *P*<0.001). At week 11, glycogen content decreased in all treatments, where the diet 3 group showed the lowest glycogen content. Significant differences were observed in diet 1 when compared with diet 2 (K-W, H=21.602, *d.f.*=3, *P*<0.001) and diet 3 (K-W, H=24.929, *d.f*.=3, *P*<0.001).

Total lipid content revealed an irregular pattern during the conditioning period, with an accentuated increase from week 0 to week 2. At week 5, total lipids decreased in oysters fed diet 1 and diet 2, while increased in oysters fed diet 4. At the end of the conditioning period, diet 1 and diet 2 groups showed a small increase and diet 4 a decrease in total lipids. Total lipid content remained almost constant in the diet 3 group from week 2 to week 11. At week 2 (T2), total lipids in diet 4 group was significantly different from all treatments, except from diet 3 (K-W, H=30.866, *d.f*.=4, *P*<0.001). At week 5, total lipids were significantly different in the unfed and diet 4 groups when compared to the other groups (ANOVA, *F*=14.729, *d.f.*=4, *P*<0.001). At the end of conditioning period, there was no significant differences between dietary groups.

In general, total energy increased during conditioning, except in the diet 3 group (25% macroalgae+75% microalgae), which showed a decrease in energy content at week 5 (T5). This decrease coincided with the lowest protein content observed. However, at week 11 diet 3 group exhibited the highest total energy content. At week 2, significant differences were observed between the diet 3 group and diets 1, 2 and 4 (ANOVA, *F*=9.581, *P*<0.001) and between the unfed group and diet 1 and 4 (ANOVA, *F*=9.581, *P*<0.001). At week 5, diet 3 group was significantly different from all other groups (K-W, H=25.438, *d.f*.=4, *P*<0.001). At the end of the conditioning period (week 11), no differences were detected between groups.

### Spearman correlation

Correlations between parameters are presented in the Supplementary data. In the unfed group (Table S1A), the condition index showed a positive correlation with glycogen content (r=0.601, *P*=0.004) and a negative correlation with total lipids (r=−0.594, *P*=0.003). On the other hand, total lipid and protein content were positively correlated (r=0.447, *P*=0.005) and both were strongly correlated with total energy (r=0.733, *P*<0.001; r=0.898, *P*<0.001, respectively). Glycogen and total lipid content were negatively correlated (r=−0.605, *P*<0.001). Condition index of oysters fed with 100% macroalgae (diet 1) (Table S1B) showed a negative correlation with protein content (r=−0.733, *P*<0.001) and with total energy (r=−0.630, *P*=0.002), and a positive correlation with glycogen content (r=0.739, *P*<0.001). Protein content exhibited a positive correlation with total lipids and total energy (r=0.359, *P*=0.012; r=0.834, *P*<0.001, respectively), while glycogen and protein content demonstrated a negative correlation (r=−0.700, *P*<0.001). Total lipids and total energy were positively correlated (r=0.693, *P*<0.001).

For the remaining diets, the condition index did not show a correlation with the other parameters (Tables S1C, S1D, S1E). In oysters conditioned with diets 2, 3 and 4, protein content was positively correlated with total lipids (r=0.452, *P*=0.001; r=0.668, *P*<0.001; r=0.628, *P*<0.001) and with total energy (r=0.937, *P*<0.001; r=0.926, *P*<0.001; r=0.918, *P*<0.001, respectively). Glycogen content was negatively correlated with protein content (r=−0.834, *P*<0.001; r=−0.731, *P*<0.001; r=−0.669, *P*<0.001, respectively), with total lipids (r=−0.460, *P*<0.001; r=−0.437, *P*=0.001; r=−0.656, *P*<0.001, respectively) and with total energy (r=−0.762, *P*<0.001; r=−0.633, *P*<0.001; r=−0.574, *P*<0.001, respectively). In diet groups 2, 3 and 4, total lipids and total energy showed a positive correlation (r=0.648, *P*<0.001; r=0.789, *P*<0.001; r=0.859, *P*<0.001, respectively).

### Digestive enzymes

Digestive enzymes activities (amylase and lipase) are presented in Table 3. Amylase activity decreased over the conditioning period for all the dietary treatments. The highest amylase activity was observed at the beginning of the conditioning period, whereas the lowest activity was observed in oysters fed with diet 2 (50% macroalgae+50% microalgae). Significant differences in amylase activity were only observed between diet 2 and diet 4 groups [Student's *t*-test, t (10)=−2.592, *P*=0.027].

**Table 3.**
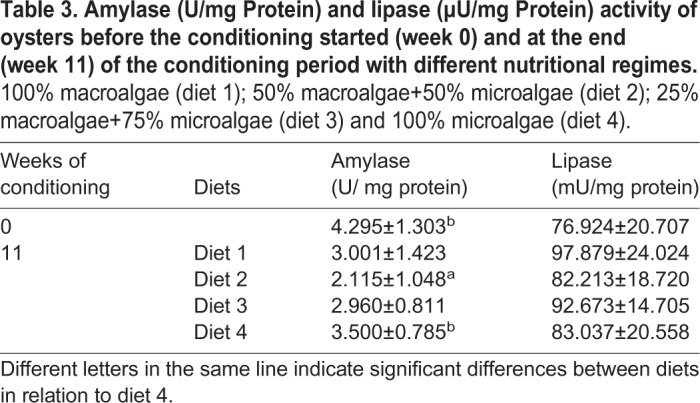
**Amylase (U/mg Protein) and lipase (µU/mg Protein) activity of oysters before the conditioning started (week 0) and at the end (week 11) of the conditioning period with different nutritional regimes.** 100% macroalgae (diet 1); 50% macroalgae+50% microalgae (diet 2); 25% macroalgae+75% microalgae (diet 3) and 100% microalgae (diet 4).

Lipase activity also showed an increase from week 0 to week 11 in all dietary groups. Oysters fed with 100% macroalgae showed the highest increase in lipase activity, followed by oysters conditioned with 25% macroalgae+75% macroalgae. Oysters conditioned with 100% microalgae exhibited the lowest lipase activity at the end of the conditioning period. No significant differences were observed in lipase activity.

### Spawning and larval rearing

Spawning success and larval parameters are expressed in Table 4. It was observed that there was a variation in the percentage of spawning individuals as a result of the dietary treatment, varying from 0% to 80%. Oysters fed diet 3 and diet 4 showed the highest percentage of spawning individuals, while the lowest performance was observed in oysters conditioned with 100% macroalgae (diet 1), with no spawning individuals. In all treatments, spawning females were represented in a higher number than males, except in the unfed group, where the opposite pattern was observed.

**Table 4.**
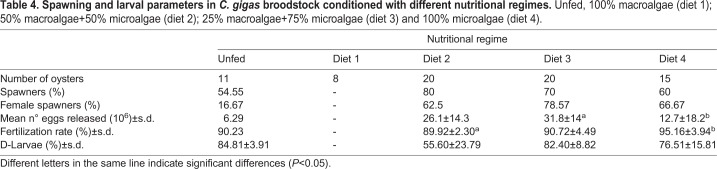
**Spawning and larval parameters in *C. gigas* broodstock conditioned with different nutritional regimes.** Unfed, 100% macroalgae (diet 1); 50% macroalgae+50% microalgae (diet 2); 25% macroalgae+75% microalgae (diet 3) and 100% microalgae (diet 4).

The number of eggs released by females ranged between 6.29 to 31.8 million, with the highest number of eggs being released by individuals conditioned with diet 3 (25% macroalgae+75% microalgae) whereas the unfed group and diet 4 group exhibited the lowest number of released eggs. Since only one female spawned in the unfed group, it was not possible to compare with the remaining groups. Therefore, the only significant differences were detected between oysters fed diet 3 and diet 4 [Student's *t*-test, t (15)=2.413, *P*=0.029].

Fertilization rate was similar between all treatments, with oysters conditioned with diet 4 exhibiting a higher fertilization rate, while individuals in diet 2 group exhibited the lowest fertilization rate. These two groups were significantly different from each other [Student's *t*-test, t (10)=−2.811, *P*=0.018].

Development veliger was similar between the unfed group and diet 3 group (84.81 and 82.40, respectively), which demonstrated the highest percentage of D-larvae. The lowest percentage was observed in oysters conditioned with diet 2 (50% macroalgae+50% microalgae). However, no significant differences were observed between treatments.

## DISCUSSION

The availability and quality of food provided during broodstock conditioning, along with other factors such as temperature, salinity and photoperiod, strongly influence the success of the conditioning and consequently the outcome of hatchery phase (González-Araya et al., 2011; Utting and Millican, 1997). In the current study, the unfed group showed a high mortality rate (72%, data not shown) during the conditioning period, which hampered the analyses of samples from week 11. It is thought that unfed oysters cannot survive when they deplete their nutritional reserves with a critical survival limit under unfed conditions from day 70 onwards (Numaguchi, 1995). In addition, Numaguchi (1995) observed an alteration of several parameters, including a drop in the glycogen content of the adductor muscle during the first week of the unfed period, an increase in weight losses and a decrease in condition index, which, altogether, may have caused high mortalities. The current study demonstrated the effect of partial and total dietary substitution of live microalgae by dry macroalgae in conditioning of *C. gigas* and the subsequent effects in physiological condition and in the reproductive outcome. Gametogenesis, biochemical composition, energy storage and spawning success were influenced by the nutritional value of the diet, as evidenced by the differences in reproductive effort.

The experimental diets were constituted by the same micro and macroalgae species and presented in equal quantities in terms of organic weight, although differing in macroalgae percentage and consequently in terms of nutritional value of the diets.

Microalgae are typically characterized by high protein content, followed by lipids and carbohydrates (Brown et al., 1997; Spolaore et al., 2006) while *U. rigida* has high carbohydrate and lipid contents (Satpati and Pal, 2011). In this work, total carbohydrates are the main constituent of all diets, which was expected in the case of the diet of 100% macroalgae (diet 1) and unexpected in the diets constituted only by microalgae (diet 4), Nevertheless, the biochemical composition of microalgae can vary highly with culture conditions and with the nutritional value of each species (Brown et al., 1997), effectively the biochemical composition of each microalgae species, when individually analyzed, showed high contents of carbohydrates (data not shown).

The seasonal biochemical cycles (energy storage and utilization) that are experienced by marine bivalves are closely related to sexual maturation (Mathieu and Lubet, 1993; Ojea et al., 2004). Gonadal development as well as condition index are considered key parameters of the sexual maturation process (Ojea et al., 2004; Walne and Mann, 1975). Gonadal development may be affected by several factors such temperature, photoperiod and nutrition. In this study, gonadal development was clearly affected by the diet provided. According to Anjos et al. (2017), the microalgae blend consisting of diatoms elicited a faster gonadal development, whereas González-Araya et al. (2011) suggested that the flagellate *I. galbana* clone T-ISO was in an intermediate position and when combined with a diatom could represent an efficient diet. Thus, in this work the microalgae blend should be able to cause an enhancement in gonadal development. The oysters conditioned with diets with higher percentages (>75%) of microalgae (diet 3 and diet 4) exhibited faster maturation during the conditioning, while a slower gonadal development was observed in oysters deprived of food and those fed with high percentage of macroalgae (>50%).

Physiological condition of the individuals assessed by condition index is closely related with gonadal development. Indeed, we observed that the condition index reflects the stage of gonadal development. For instance, at week 2 of conditioning the regression in gonadal development coincides with a decline in condition index for all experimental diets. Oysters conditioned with diet 3 and diet 4 (75% and 100% microalgae, respectively) exhibited a better physiological condition, whereas individuals fed with 100% macroalgae (diet 1) showed a weaker physiological condition. High levels of macroalgae directly affects the physiological condition of oysters, although lower percentages of dietary inclusion of macroalgae (25%) elicited similar results to those fed with 100% microalgae. Thus, broodstock oysters fed with diet 3 and diet 4 had an optimal nutritional regime, since somatic and reproductive processes were satisfied. On the other hand, oysters deprived of food and those fed with 100% macroalgae (diet 1) probably adjusted their metabolic needs and channeled the energy for maintenance of the basal metabolism. According to Albentosa et al. (2007), one of the main effects of starvation or inadequacy of quality and quantity of food provided in invertebrates is a decrease in metabolism down to maintenance levels. The slow evolution in gonadal maturation and the regression in condition index of unfed oysters and oysters fed with diet 1 may suggested that, in case of nutritional stress, reproduction and survival seem to be priorities, thus available energy is allocated to ensure the viability of the species or survival of the individual (Anjos et al., 2017; Joaquim et al., 2011).

When food is nutritionally balanced and abundant, energy is stored prior to gametogenesis in the form of protein, glycogen and lipids (Ojea et al., 2004). The energy is used to synthesize gametes which are released during spawning (Albentosa et al., 2007; Joaquim et al., 2011).

In bivalves, glycogen and total lipids are the main reserves for gametogenesis while proteins are mainly used in structural function (Anjos et al., 2017; Matias et al., 2016). According to Pogoda et al. (2013), during gametogenesis, glycogen is the preferential form of energy reserve in oysters. After an initial period of storage, glycogen is simultaneously used with food as an energy support for gametogenesis (González-Araya et al., 2011). In all experimental diets, proteins were used as energy for maintenance, while glycogen was used as energy source for gametogenesis. In general, during conditioning, glycogen content was negatively correlated with lipid and protein content; a decrease in glycogen content was followed by an increase in lipid and protein levels. Several authors have described the negative correlation between glycogen and lipids (Beninger and Lucas, 1984; Ojea et al., 2004), probably due to glycogen conversion to lipids biosynthesized during gamete formation (Gabbott, 1975). Broodstock oysters conditioned with diet 3 (25% macroalgae+75% microalgae) and diet 4 (100% microalgae) showed a better physiologic condition as well as successful gonadal maturation and low levels of glycogen and high lipid content, which are the main reserve of oocytes (Soudant et al., 1999; Utting and Millican, 1997).

In the current study, the diet formulation influenced the use of energy reserves of the oysters. Food deprivation and 100% macroalgae diet had a direct effect on biochemical composition and consequently on physiological condition. Both groups showed an irregular pattern in glycogen and total lipid content, indicating that the animals channeled energy reserves in a different way to compensate for the lack in nutritional supply. Moreover, it has been suggested that proteins are used as main energy source in situations of nutritional stress and when carbohydrates reserves have already been depleted (Albentosa et al., 2007; Beninger and Lucas, 1984; Joaquim et al., 2011). In this case, the negative correlation observed between proteins and condition index in diet 1 group (100% macroalgae) may indicate that these individuals were in a situation of physiological stress, thus resorting to alternative energy resources.

In addition to the decrease in glycogen content and the increase in total lipids content between week 0 and week 11 of the conditioning period, a decrease in the activity of digestive enzymes was also observed. From the beginning to the end of the conditioning period, amylase activity decreased in all experimental diets, regardless of the dietary treatment. Due to amylase key role in carbohydrate digestion, a reduction in amylase activity may cause a reduction in glucose availability (LeMoine et al., 1997; Sellos et al., 2003), and consequently a reduction in glycogen deposition. Similarly, with the increase of total lipid content at the end of the conditioning period, an increase in lipase activity in hepatopancreas was expected. Amylase activity in oysters fed diet 2 (50% macroalgae+50% microalgae) was significantly lower than in oysters fed diet 4 (100% microalgae), which may indicate an inhibitory effect of *U. rigida* on amylase activity. Such inhibitory activity has been observed in previous studies with red, brown and green macroalgae (Admassu et al., 2018; Heo et al., 2005; Kim et al., 2014) and may be correlated with bioactive tannins in the macroalgae (de Oliveira et al., 2009). The amylase inhibition highlights the need to limit macroalgae content in oyster diet. In the current study, oysters fed diet 3 appear to be the closest to the ideal diet formulation, with similar digestive enzyme activity as the traditional 100% microalgae. The need to limit macroalgae supplementation levels to about 20% of the diet has been previously observed by Xuan et al. (2013) and Al-Asgah et al. (2016).

The success of the conditioning was evaluated by the reproductive outcome, which considered spawning success, fecundity, fertilization rate and development of D-veliger larvae.

Several experiments suggested that reproductive success is influenced by the quality of food provided to broodstock oysters during conditioning (Millican and Helm, 1994; Nevejan et al., 2003; Utting and Millican, 1998). In fact, the conditioning diets had a clear effect on the reproductive success of oysters in the current study. When oysters were fed with 100% macroalgae, spawning did not occur. This may be due to the fact that oysters fed 100% macroalgae may channel all available energy to survival instead reproduction, which is commonly observed in animals under severe nutritional stress. That was also the case with unfed oysters, although, unfed oysters allocated all available energy to reproduction. The best reproductive outcome was obtained in oysters conditioned with diet 3 (75% macroalgae+25% microalgae) and diet 4 (100% microalgae), with both groups showing similar responses. Nevertheless, diet 3 showed better development in D-veliger larvae than oysters fed diet 4, which is a well-established diet for oyster conditioning.

In conclusion, the replacement of 25% of microalgae with dry *U. rigida* may lead to similar reproductive success, nutritional quality and physiological condition of broodstock oysters fed 100% live microalgae. Conversely, 100% macroalgae substitution had a negative impact in broodstock conditioning while better results during conditioning were obtained with a partial replacement of live microalgae (25%). Our conclusions are in agreement with previous studies which have reported that a replacement of live microalgae is indeed possible, however, only a partial substitution has been proven to be successful (Arney et al., 2015; Boeing, 1997; Camacho et al., 2004; Langdon and Önal, 1999; Pérez Camacho et al., 2007; Tanyaros and Chuseingjaw, 2016). The use of an alternative diet (25% macroalgae+75% microalgae) to the traditional 100% microalgae is beneficial during the conditioning period of *C. gigas* and represents an economic advantage for bivalve hatcheries, since it allows them to minimize operation costs.

## MATERIALS AND METHODS

### Experimental design

#### Culture of microalgae and diet formulation

Microalgae *Isochrysis* aff. *galbana* (T.ISO), *S. costatum* (SKT) and *Chaetoceros calcitrans* (C-Cal) were batch-cultured in a plastic bag (80 L) with filtered (0.35 µm) UV-treated seawater (salinity 33). Seawater was chlorinated for 24 h, neutralized with thiosulphate and enriched with f/2 medium before inoculation. Microalgae were grown at a temperature of 18±2°C, under continuous aeration, to improve growth and avoid algae settlement, and under constant conditions of light, at an intensity of 9.900 lux at the culture surface. Microalgae were harvested daily in the late-exponential growth phase.

Before feeding the animals, algal densities were determined daily with a standard algal cell counts (Bürker chamber).

Five nutritional regimes were tested, of which three were formulated with different proportions of commercial dry macroalgae *U. rigida* (<150 µm) (acquired from enterprise Algaplus, Ílhavo, Portugal): diet 1–100% macroalgae, diet 2–50% macroalgae and 50% of microalgae mix and diet 3–25% macroalgae and 75% microalgae mix; diet 4-100% microalgae mix (positive control) and an unfed group as a negative control.

Microalgae mix was formulated with one third T.ISO (size: 3×5 µm; dry weight: 30.5 pg) and two thirds diatoms: 75% of SKT (size: 10×5 µm; dry weight: 52.2 pg) and 25% of C-Cal (size: 3–6 µm; dry weight: 11.3 pg) (Brown et al., 1997).

#### Broodstock conditioning

900 adult oysters from Ria de Aveiro (Portugal, western coast; 40°42′N; 08°40′W) were equal and randomly distributed into five groups, and each group was conditioned with one nutritional regime.

For each nutritional regime tested, triplicate tanks were set up and each group of oysters (83.45±12.42 g total mean weight; 2.40±0.77 g average dry meat weight; 10.4±0.96 cm mean length) was randomly distributed in the tanks (25 L).

Experimental tanks contained natural seawater filtered through 0.35 µm in a flow-through system at a flow rate of 0.8 L min^−1^. Water salinity was 33 and water temperature was maintained at 21±1°C by using heat exchangers with titanium plates.

According to the macroalgae percentage in each diet, macroalgae were weighed, re-suspended in natural filtered seawater and posteriorly added to the food supply tanks, with strong and continuous aerations to avoid deposition.

Food was added daily to the tanks with a pump, at a ratio of 4% of oyster dry weight (g) in algal dry weight (mg) (Delaporte et al., 2006; Helm et al., 2004; Utting and Millican, 1997).

In order to keep food rations constant, the amount of food was adjusted daily according to the total biomass in each experimental condition.

*C. gigas* broodstock were conditioned during a period of 11 weeks, from February 2017 to April 2017.

During the conditioning period, samplings were performed at the beginning of the experiment (week 0) and at weeks 2, 5 and 11 (end of the trial).

At each sample time, three groups of ten oysters were randomly selected for condition index, biochemical composition (proteins, total lipids and glycogen content) and histological analysis of gonadal development. At week 11, the unfed group was not sampled due to the high mortality rate.

At weeks 0 and 11, oysters from each treatment were sampled and stored at −80°C for digestive enzyme analysis. Samples for each treatment were stored as well at −20°C for condition index and biochemical composition analyses.

Diets were sampled for nutritional composition (proteins, carbohydrates and total lipids), centrifuged and stored at −80°C for further analysis.

#### Spawning and larval rearing

At the end of the conditioning period (week 11), the remaining oysters from each treatment were placed into the spawning tanks (unfed, *N*=11; diet 1, *N*=8; diet 2, *N*=20; diet 3, *N*=20; diet 4, *N*=15) and were induced to spawn. Spawning was triggered by thermal stimulation, through a rapid increase in water temperature from 15°C to 30±1°C at 2 h intervals. To avoid polyspermia, individuals that showed a response to the stimulus were separated into individual receptacles. Fecundity was evaluated through counting three 50 µl samples taken from each oocyte suspension of each female. Oocytes from each female were fertilized by adding a sperm mixture from all males from the same diet, in a ratio of 1:10 oocyte/spermatozoa in a visual field of microscope (Matias et al., 2009). After 1 h of fertilization, three 50 µl samples were taken to assess fertilization rate. Embryos from each female were incubated at 22°C in triplicate 1 L recipients, with 0.35 µm filtered and UV-irradiated seawater, at a density of 100 eggs per milliliter.

At the end of 42 h of incubation, D-larvae were collected by sieving through a 40 µm mesh screen, and the percentage of D-larvae (veliger rate) was calculated relative to initial number of embryos.

### Histology

Individuals were opened and visceral tissue was excised and fixed in Davidson's solution (Shaw and Battle, 1957) for at least 48 h. Thereafter, samples were washed in tap water for 30 min and then transferred to ethanol (70%).

Tissues were dehydrated with a series of increasing concentration ethanol treatments and included in paraffin. Seven micrometer sections were cut, mounted on glass slides and stained with hematoxylin-eosin (Martoja and Martoja, 1967).

Sections were analyzed under an optical microscope for sex determination (male, female or hermaphrodite) and gonadal development stage evaluation. Gonadal stages were classified in five stages of development: stage 0, resting; stage I, early gametogenesis; stage II, late gametogenesis; stage III, maturation; stage IV, spawning and reabsorbing ­– according to what was described by Mann (1979). Whenever more than one stage was observed in one single section, the decision of staging criteria was based upon the most representative stage of the preparation.

### Condition index

After opening each individual, soft tissues were removed and placed on an absorbent paper to drain for 5 min. Both shell and soft tissue were then dried at 80°C and weighed after 24 h. Then, dried meat was turned into ashes in a muffle furnace at 450°C for 24 h and reweighed.

Condition index was calculated as a relation between ash-free dry weight of meat (g) and shell dry weight (g), as described by Walne and Mann (1975): [ash-free dry weight (AFDW) of meat (g)/shell dry weight (g)]×100.

### Biochemical composition analysis

#### Nutritional compositions of diets

Nutritional composition of different diets was analyzed in triplicate for each parameter. Kjeldahl assay based on 990.03 AOAC was used for protein determination and Soxhlet extraction method based on 945.16 AOAC for total lipids determination. Moisture was assayed by infrared drying at 105°C (Scaltec SMO 01, Heiligenstadt, Germany) until constant weight and expressed as g of moisture per 100 g of sample. Total carbohydrate content was determined by difference and was calculated using the following formula:

100–(weight (g) [protein+fat+water+ash] in 100 g of sample).

#### Proximal biochemical analysis of broodstock

For each diet, oysters previously sampled and stored at −20°C were defrosted and opened. The entire soft body was separated from the shell and homogenized in an ice bath. Proteins, glycogen and total lipid content were determined by standard methods. The modified Lowry method (Shakir et al., 1994) was used to determined protein content, after extraction with normal sodium hydroxide. Glycogen content was determined from dried homogenate (80°C for 24 h) using anthrone as reagent (Viles and Silverman, 1949). Total lipid content was extracted from fresh homogenate in chloroform/methanol (Folch et al., 1957) and estimated spectrophotometrically after charring with concentrated sulphuric acid (Marsh and Weinstein, 1966). Biochemical composition results are expressed as a total organic ash-free dry weight (µg mg^−1^ of AFDW).

Energy content was calculated using the energy equivalents for proteins (17.9 KJ g^−1^), glycogen (17.2 KJ g^−1^) and total lipids (33 KJ g^−1^) (Beukema and De Bruin, 1979, Paine, 1971, Beninger and Lucas, 1984, respectively). Results are expressed as KJ mg^−1^AFDW.

#### Digestive enzymes analyses

Hepatopancreas were homogenized in 1:10 of extraction buffer (50 mM Tris HCl and 200 mM NaCl, pH 8). After being centrifuged at 7000 ***g*** for 30 min at 4°C, homogenates were aliquoted (200 µl) for further analysis of protein, lipase and amylase. Aliquots were stored at −80°C until analysis.

An initial protein quantification was needed since amylase and lipase digestive enzymes were calculated in relation to the amount of protein in the tissue.

Protein quantification was performed following the folin-phenol method, according to Lowry et al. (1951).

Amylase activity was measured from the increase in reducing maltose by the hydrolysis of α-D (1, 4) glucosidic bond in polysaccharides and stained with 3, 5-dinitrosalicylic acid (DNS), as described by Bernfeld (1951). The method used was the Areekijseree et al. (2004) modified method. Final unit is expressed as enzymatic unit per protein mg.

Lipase activity was performed by using ρ-nitrophenyl substrate as described by Winkler and Stuckmann (1979). The ρ-nitrophenyl formation was then quantified by absorbance reading at 410 nm. Final unit is expressed as micro units per milligram of protein (µU/mg protein).

### Statistical analysis

Results are expressed as mean±s.d. (s.d.). Depending on the violation or not of the normality and homogeneity of variance assumptions, one-way analysis of variance (ANOVA) or Kruskal–Wallis nonparametric tests were applied to compare condition index and biochemical composition of diets and broodstock among nutritional regimes. These analyses were performed separately for each sampling time (2, 5 and 11 weeks of conditioning period). Whenever applicable, Tukey's, Dunn's or Tamhane's T2 post-hoc tests (depending validation of normality and homogeneity of variance assumptions) were applied to identify the differences.

For each diet, Spearman Rank Order correlation was used to determine the degree of association between parameters (condition index, protein, glycogen, total lipids and total energy).

Statistical differences regarding to digestive enzymes results, as well as in the number of eggs released, fertilization and D-veliger larvae development were determined by performing a parametric Student's *t-*test, comparing all diets with diet 4 (positive control). Significance level was set as *P*≤0.05 for all statistical tests. Statistical analyses were undertaken using Sigmaplot 12.5 statistical package.

## Supplementary Material

Supplementary information

First Person interview

## References

[BIO035923C1] Abdel-Warith A. W. A., Younis E. S. M. I. and Al-Asgah N. A. (2016). Potential use of green macroalgae Ulva lactuca as a feed supplement in diets on growth performance, feed utilization and body composition of the African catfish, Clarias gariepinus. *Saudi J. Biol. Sci.* 23, 404-409. 10.1016/j.sjbs.2015.11.010 27081367 PMC4818333

[BIO035923C2] Admassu H., Gasmalla M. A. A., Yang R. and Zhao W. (2018). Evaluation of the in vitro α-amylase enzyme inhibition potential of commercial dried laver (Porphyra Species) seaweed protein hydrolysate. *Turkish J. Fish. Aquat. Sci.* 18, 547-556.

[BIO035923C3] Al-Asgah N. A., Younis E.-S. M., Abdel-Warith A.-W. A. and Shamlol F. S. (2016). Evaluation of red seaweed Gracilaria arcuata as dietary ingredient in African catfish, Clarias gariepinus. *Saudi J. Biol. Sci.* 23, 205-210. 10.1016/j.sjbs.2015.11.006 26981001 PMC4778581

[BIO035923C4] Albentosa M., Fernández-Reiriz M. J., Labarta U. and Pérez-Camacho A. (2007). Response of two species of clams, Ruditapes decussatus and Venerupis pullastra, to starvation: Physiological and biochemical parameters. *Comp. Biochem. Physiol. B Biochem. Mol. Biol.* 146, 241-249. 10.1016/j.cbpb.2006.10.109 17196861

[BIO035923C5] Anjos C., Baptista T., Joaquim S., Mendes S., Matias A. M., Moura P., Simões T. and Matias D. (2017). Broodstock conditioning of the Portuguese oyster (Crassostrea angulata, Lamarck, 1819): influence of different diets. *Aquac. Res.* 48, 3859-3878. 10.1111/are.13213

[BIO035923C6] Areekijseree M., Engkagul A., Kovitvadhi U., Thongpan A., Mingmuang M., Pakkong P. and Rungruangsak-Torrissen K. (2004). Temperature and pH characteristics of amylase and proteinase of adult freshwater pearl mussel, Hyriopsis (Hyriopsis) bialatus Simpson 1900. *Aquaculture* 234, 575-587. 10.1016/j.aquaculture.2003.12.008

[BIO035923C7] Arney B., Liu W., Forster I. P., McKinley R. S. and Pearce C. M. (2015). Feasibility of dietary substitution of live microalgae with spray-dried Schizochytrium sp. or Spirulina in the hatchery culture of juveniles of the Pacific geoduck clam (Panopea generosa). *Aquaculture* 444, 117-133. 10.1016/j.aquaculture.2015.02.014

[BIO035923C8] Bautista-Teruel M. N., Millamena O. M. and Fermin A. C. (2001). Reproductive performance of hatchery-bred donkey's ear abalone, Haliotis asinina, Linne, fed natural and artificial diets. *Aquac. Res.* 32, 249-254. 10.1046/j.1355-557x.2001.00022.x

[BIO035923C9] Beninger P. G. and Lucas A. (1984). Seasonal variations in condition, reproductive activity, and gross biochemical composition of two species of adult clam reared in a common habitat: Tapes decussatus L. (Jeffreys) and Tapes philippinarum (Adams & Reeve). *J. Exp. Mar. Bio. Ecol.* 79, 19-37. 10.1016/0022-0981(84)90028-5

[BIO035923C10] Bernfeld P. (1951). Enzymes of starch degradation and synthesis. *Adv. Enzymol. Relat. Subj. Biochem.* 12, 379-428. 10.1002/9780470122570.ch7 14885023

[BIO035923C11] Beukema J. J. and De Bruin W. (1979). Calorific values of the soft parts of the tellinid bivalve Macoma balthica (L.) as determined by two methods. *J. Exp. Mar. Bio. Ecol.* 37, 19-30. 10.1016/0022-0981(79)90023-6

[BIO035923C12] Bilbao A., Uriarte I., del Pino Viera M., Sosa B., Fernández-Palacios H. and Hernández-Cruz C. M. (2012). Effect of macroalgae protein levels on some reproductive aspects and physiological parameters for the abalone, haliotis tuberculata coccinea (Reeve 1846). *J. World Aquac. Soc.* 43, 764-777. 10.1111/j.1749-7345.2012.00617.x

[BIO035923C13] Boeing P. (1997). Use of spray-dried Schizochytrium sp. as a partial algal replacement for juvenile bivalves. *J. Shellfish Res* 16, 284.

[BIO035923C14] Borowitzka M. A. (1997). Microalgae for aquaculture: opportunities and constraints. *J. Appl. Phycol.* 9, 393-401. 10.1023/A:1007921728300

[BIO035923C15] Boudry P., Heurtebise S., Collet B., Cornette F. and Gérard A. (1998). Differentiation between populations of the Portuguese oyster, Crassostrea angulata (Lamark) and the Pacific oyster, Crassostrea gigas (Thunberg), revealed by mtDNA RFLP analysis. *J. Exp. Mar. Biol. Ecol.* 226, 279-291. 10.1016/S0022-0981(97)00250-5

[BIO035923C16] Brown M. R. (2002). Nutritional value of microalgae for aquaculture. In: Cruz-Suarez, L. E., Ricque-Marie, D., Tapia-Salazar, M., Gaxiola-Cortes, M. G., Simoes, N. (eds). Avances en nutricion acuicola VI. Memorias del VI SymposiumInternacional de Nutricion Acuicola. 3–6th September, Cancun, Mexico.

[BIO035923C17] Brown M. and Robert R. (2002). Preparation and assessment of microalgal concentrates as feeds for larval and juvenile Pacific oyster (Crassostrea gigas). *Aquaculture* 207, 289-309. 10.1016/S0044-8486(01)00742-6

[BIO035923C18] Brown M. R., Jeffrey S. W., Volkman J. K. and Dunstan G. A. (1997). Nutritional properties of microalgae for mariculture. *Aquaculture* 151, 315-331. 10.1016/S0044-8486(96)01501-3

[BIO035923C19] Camacho P., Salinas J. M., Fuertes C. and Delgado M. (2004). Preparation of single cell detritus from Laminaria saccharina as a hatchery diet for bivalve mollusks. *Mar. Biotechnol.* 6, 642-649. 10.1007/s10126-004-2901-z 15747094

[BIO035923C20] Cook E. J. and Kelly M. S. (2007). Effect of variation in the protein value of the red macroalga Palmaria palmata on the feeding, growth and gonad composition of the sea urchins Psammechinus miliaris and Paracentrotus lividus (Echinodermata). *Aquaculture* 270, 207-217. 10.1016/j.aquaculture.2007.01.026

[BIO035923C21] Coutteau P. and Sorgeloos P. (1992). The use of algal substitues and the requirement for live algae in the hatchery and nursery rearing of bivalve molluscs: an international survey. *J. Shellfish Res.* 11, 467-476.

[BIO035923C22] Delaporte M., Soudant P., Lambert C., Moal J., Pouvreau S. and Samain J.-F. (2006). Impact of food availability on energy storage and defense related hemocyte parameters of the Pacific oyster Crassostrea gigas during an experimental reproductive cycle. *Aquaculture* 254, 571-582. 10.1016/j.aquaculture.2005.10.006

[BIO035923C23] Delgado M., Pérez Camacho A., Labarta U. and Fernández-Reiriz M. J. (2004). The role of lipids in the gonadal development of the clam Ruditapes decussatus (L.). *Aquaculture* 241, 395-411. 10.1016/j.aquaculture.2004.07.018

[BIO035923C24] de Oliveira M. N., Freitas A. L. P., Carvalho A. F. U., Sampaio T. M. T., Farias D. F., Alves Teixeira D. I., Gouveia S. T., Pereira J. G. and Sena M. M. C. C. (2009). Nutritive and non-nutritive attributes of washed-up seaweeds from the coast of Ceará, Brazil. *Food Chem.* 115, 254-259. 10.1016/j.foodchem.2008.12.004

[BIO035923C25] Ergün S., Soyutürk M., Güroy B., Güroy D. and Merrifield D. (2009). Influence of Ulva meal on growth, feed utilization, and body composition of juvenile Nile tilapia (Oreochromis niloticus) at two levels of dietary lipid. *Aquac. Int.* 17, 355-361. 10.1007/s10499-008-9207-5

[BIO035923C26] Fabioux C., Huvet A., Le Souchu P., Le Pennec M. and Pouvreau S. (2005). Temperature and photoperiod drive Crassostrea gigas reproductive internal clock. *Aquaculture* 250, 458-470. 10.1016/j.aquaculture.2005.02.038

[BIO035923C27] Fleurence J. (1999). Seaweed proteins: biochemical, nutritional aspects and potential uses. *Trends Food Sci. Technol.* 10, 25-28. 10.1016/S0924-2244(99)00015-1

[BIO035923C28] Fleurence J., Morançais M., Dumay J., Decottignies P., Turpin V., Munier M., Garcia-Bueno N. and Jaouen P. (2012). What are the prospects for using seaweed in human nutrition and for marine animals raised through aquaculture? *Trends Food Sci. Technol.* 27, 57-61. 10.1016/j.tifs.2012.03.004

[BIO035923C29] Folch J., Lees M. and Stanley G. H. S. (1957). A simple method for the isolation and purification of total lipides from animal tissues. 55, 999-1033. 13428781

[BIO035923C30] Gabbott P. (1975). Storage cycles in marine bivalve molluscs: a hypothesis concerning the relationship between glycogen metabolism and gametogenesis. In *Proceedings of Ninth European Marine Biology Symposium* (ed. Barnes H.), pp. 191-211. Aberdeen: Aberdeen University Press.

[BIO035923C31] González-Araya R., Quéau I., Quéré C., Moal J. and Robert R. (2011). A physiological and biochemical approach to selecting the ideal diet for Ostrea edulis (L.) broodstock conditioning (part A). *Aquac. Res.* 42, 710-726. 10.1111/j.1365-2109.2010.02731.x

[BIO035923C32] González-Araya R., Lebrun L., Quéré C. and Robert R. (2012). The selection of an ideal diet for Ostrea edulis (L.) broodstock conditioning (part B). *Aquaculture* 362–363, 55-66. 10.1016/j.aquaculture.2012.06.029

[BIO035923C33] Guedes A. C. and Malcata F. X. (2012). Chapter 4 – Nutritional Value and Uses of Microalgae in Aquaculture. *Muchlisin, Z. (Ed.), ISBN: 978-953-307-974-5*, InTech.

[BIO035923C34] Helm M., Holand D. L. and Stephenson R. R. (1973). The effect of supplementary algal feeding of a hatchery breeding stock of ostrea Edulis L. on larval vigour. *J. Mar. Biol. Assoc. UK* 53, 673-684. 10.1017/S0025315400058872

[BIO035923C35] Helm M. M., Bourne N. and Lovatelli A. (2004). Hatchery culture of bivalves. A practical manual. In: *FAO* Fisheries Technical Paper, Vol. 471 (ed. by A. Lovatelli), pp. 1–177. Food and Agriculture Organization of the United Nations Publishing, Rome, Italy.

[BIO035923C36] Heo S. J., Park E. J., Lee K. W. and Jeon Y. J. (2005). Antioxidant activities of enzymatic extracts from brown seaweeds. *Bioresour. Technol.* 96, 1613-1623. 10.1016/j.biortech.2004.07.013 15978995

[BIO035923C37] Joaquim S., Matias D., Matias A. M., Moura P., Arnold W. S., Chícharo L. and Baptista Gaspar M. (2011). Reproductive activity and biochemical composition of the pullet carpet shell *Venerupis senegalensis* (Gmelin, 1791) (Mollusca: Bivalvia) from Ria de Aveiro (northwestern coast of Portugal). *Sci. Mar.* 75, 217-226. 10.3989/scimar.2011.75n2217

[BIO035923C38] Kemp J. O. G., Britz P. J. and Toledo Agüero P. H. (2015). The effect of macroalgal, formulated and combination diets on growth, survival and feed utilisation in the red abalone Haliotis rufescens. *Aquaculture* 448, 306-314. 10.1016/j.aquaculture.2015.06.016

[BIO035923C39] Kheder R. B., Quéré C., Moal J. and Robert R. (2010). Effect of nutrition on Crassostrea gigas larval development and the evolution of physiological indices. Part A: quantitative and qualitative diet effects. *Aquaculture* 305, 165-173. 10.1016/j.aquaculture.2010.04.022

[BIO035923C40] Kim K.-T., Rioux L.-E. and Turgeon S. L. (2014). Alpha-amylase and alpha-glucosidase inhibition is differentially modulated by fucoidan obtained from Fucus vesiculosus and Ascophyllum nodosum. *Phytochemistry* 98, 27-33. 10.1016/j.phytochem.2013.12.003 24388677

[BIO035923C41] Knauer J. and Southgate P. C. (1999). A review of the nutritional requirements of bivalves and the development of alternative and artificial diets for bivalve aquaculture. *Rev. Fish. Sci.* 7, 241-280. 10.1080/10641269908951362

[BIO035923C42] Langdon C. and Önal E. (1999). Replacement of living microalgae with spray-dried diets for the marine mussel Mytilus galloprovincialis. *Aquaculture* 180, 283-294. 10.1016/S0044-8486(99)00197-0

[BIO035923C43] LeMoine S., Sellos D., Moal J., Daniel J. Y., Serrano F. S. R., Samain J. F. and VanWormhoudt A. (1997). Amylase in Pecten maximus (Mollusca, bivalves): Protein and cDNA characterization; quantification of the expression in the digestive gland. *Mol. Mar. Biol. Biotechnol.* 6, 228-237. 9284561

[BIO035923C44] Lowry O. H., Rosebrough N. J., Farr A. L. and Randall R. J. (1951). The folin by oliver. *J. Chem. Biol.* 193, 265-275. 14907713

[BIO035923C45] Mann R. (1979). Some biochemical and physiological aspects of growth and gametogenesis in Crassostrea gigas and Ostrea edulis grown at sustained elevated temperatures. *J. Mar. Biol. Assoc. U.K.* 59, 546-559. 10.1017/S0025315400046208

[BIO035923C46] Marsh J. B. and Weinstein D. B. (1966). Simple charring method for determination of lipids. *J. Lipid Res.* 7, 574-576. 5965305

[BIO035923C47] Marshall R., Mckinley S. and Pearce C. M. (2010). Effects of nutrition on larval growth and survival in bivalves. *Rev. Aquac.* 2, 33-55. 10.1111/j.1753-5131.2010.01022.x

[BIO035923C48] Martoja R. and Martoja M. (1967). Initiations aux techniques de l'histologie animale. *Masson Cie, Paris, France*.

[BIO035923C49] Mathieu M. and Lubet P. (1993). Storage tissue metabolism and reproduction in marine bivalves—a brief review. *Invertebr. Reprod. Dev.* 23, 123-129. 10.1080/07924259.1993.9672303

[BIO035923C50] Matias D., Joaquim S., Leitão A. and Massapina C. (2009). Effect of geographic origin, temperature and timing of broodstock collection on conditioning, spawning success and larval viability of Ruditapes decussatus (Linné, 1758). *Aquac. Int.* 17, 257-271. 10.1007/s10499-008-9197-3

[BIO035923C51] Matias D., Joaquim S., Ramos M., Sobral P. and Leitão A. (2011). Biochemical compounds’ dynamics during larval development of the carpet-shell clam Ruditapes decussatus (Linnaeus, 1758): Effects of mono-specific diets and starvation. *Helgol. Mar. Res.* 65, 369-379. 10.1007/s10152-010-0230-3

[BIO035923C52] Matias D., Joaquim S., Matias A. M. and Leitão A. (2016). Reproductive effort of the European clam Ruditapes decussatus (Linnaeus, 1758): influence of different diets and temperatures. *Invertebr. Reprod. Dev.* 60, 49-58. 10.1080/07924259.2015.1126537

[BIO035923C53] Millican P. F. and Helm M. M. (1994). Effects of nutrition on larvae production in the European flat oyster, Ostrea edulis. *Aquaculture* 123, 83-94. 10.1016/0044-8486(94)90121-X

[BIO035923C54] Muller-Feuga A. (2000). The role of microalgae in aquaculture: situation and trends. *J. Appl. Phycol.* 12, 527-534. 10.1023/A:1008106304417

[BIO035923C55] Nevejan N., Courtens V., Hauva M., Gajardo G. and Sorgeloos P. (2003). Effect of lipid emulsions on production and fatty acid composition of eggs of the scallop Argopecten purpuratus. *Marine Biology* 143, 327-338. 10.1007/s00227-003-1076-x

[BIO035923C56] Numaguchi K. (1995). Influences of unfed condition on the mortality of pearl oyster Pinctada fucata martensii. *Fish. Sci.* 61, 739-742. 10.2331/fishsci.61.739

[BIO035923C57] Ojea J., Pazos A. J., Martínez D., Novoa S., Sánchez J. L. and Abad M. (2004). Seasonal variation in weight and biochemical composition of the tissues of Ruditapes decussatus in relation to the gametogenic cycle. *Aquaculture* 238, 451-468. 10.1016/j.aquaculture.2004.05.022

[BIO035923C58] Ortiz J., Romero N., Robert P., Araya J., Lopez-Hernández J., Bozzo C., Navarrete E., Osorio A. and Rios A. (2006). Dietary fiber, amino acid, fatty acid and tocopherol contents of the edible seaweeds Ulva lactuca and Durvillaea antarctica. *Food Chem.* 99, 98-104. 10.1016/j.foodchem.2005.07.027

[BIO035923C59] Paine R. T. (1971). The measurement and application of the calorie to ecological problems. *Annu. Rev. Ecol. Syst.* 2, 145-164. 10.1146/annurev.es.02.110171.001045

[BIO035923C60] Parwadani-Aji L. (2011). The use of algae concentrates, dried algae and algal substitutes to feed bivlaves. *Makara, Sains* 15, 1-8.

[BIO035923C61] Peng Y., Hu J., Yang B., Lin X. P., Zhou X. F., Yang X. W. and Liu Y. 2015 Chemical composition of seaweeds. In: Seaweed Sustainability: Food and Non-Food Applications. Edited by Tiwari, B. K. and Troy, D. J. 79-124. Elsevier Inc., USA.

[BIO035923C62] Pérez Camacho A., Salinas J. M., Delgado M. and Fuertes C. (2007). Use of single cell detritus (SCD) produced from Laminaria saccharina in the feeding of the clam Ruditapes decussatus (Linnaeus, 1758). *Aquaculture* 266, 211-218. 10.1016/j.aquaculture.2006.12.033

[BIO035923C63] Pogoda B., Buck B. H., Saborowski R. and Hagen W. (2013). Biochemical and elemental composition of the offshore-cultivated oysters Ostrea edulis and Crassostrea gigas. *Aquaculture* 400–401, 53-60. 10.1016/j.aquaculture.2013.02.031

[BIO035923C64] Pronker A. E., Nevejan N. M., Peene F., Geijsen P. and Sorgeloos P. (2008). Hatchery broodstock conditioning of the blue mussel Mytilus edulis (Linnaeus 1758). Part I. Impact of different micro-algae mixtures on broodstock performance. *Aquac. Int.* 16, 297-307. 10.1007/s10499-007-9143-9

[BIO035923C65] Satpati G. G. and Pal R. (2011). Biochemical composition and lipid characterization of marine green alga Ulva rigida- a nutritional approach. *J. Algal Biomass Util.* 2, 10-13.

[BIO035923C66] Sellos D., Moal J., Degremont L., Huvet A., Daniel J.-Y., Nicoulaud S., Boudry P., Samain J.-F. and Van Wormhoudt A. (2003). Structure of amylase genes in populations of pacific cupped oyster (Crassostrea gigas): Tissue expression and allelic polymorphism. *Mar. Biotechnol.* 5, 360-372. 10.1007/s10126-002-0089-7 14719164

[BIO035923C67] Shakir F. K., Audilet D., Drake A. J. III and Shakir K. M. M. (1994). A rapid protein determination by modification of the lowry procedure. *Anal. Biochem.* 216, 232-233. 10.1006/abio.1994.1031 8135358

[BIO035923C68] Shaw B. L. and Battle H. I. (1957). The gross and microscopic anatomy of the digestive tract of the oyster crassostrea virginica (Gmelin). *Can. J. Zool.* 35, 325-347. 10.1139/z57-026

[BIO035923C69] Soudant P., Van Ryckeghem K., Marty Y., Moal J., Samain J. F. and Sorgeloos P. (1999). Comparison of the lipid class and fatty acid composition between a reproductive cycle in nature and a standard hatchery conditioning of the Pacific Oyster Crassostrea gigas. *Comp. Biochem. Physiol. B Biochem. Mol. Biol.* 123, 209-222. 10.1016/S0305-0491(99)00063-2

[BIO035923C70] Spolaore P., Joannis-Cassan C., Duran E. and Isambert A. (2006). Commercial applications of microalgae. *J. Biosci. Bioeng.* 101, 87-96. 10.1263/jbb.101.87 16569602

[BIO035923C71] Tanyaros S. and Chuseingjaw S. (2016). A partial substitution of microalgae with single cell detritus produced from seaweed (Porphyra haitanensis) for the nursery culture of tropical oyster (Crassostrea belcheri). *Aquac. Res.* 47, 2080-2088. 10.1111/are.12662

[BIO035923C72] Utting S. D. and Millican P. F. (1997). Techniques for the hatchery conditioning of bivalve broodstocks and the subsequent effect on egg quality and larval viability. *Aquaculture* 155, 45-54. 10.1016/S0044-8486(97)00108-7

[BIO035923C73] Utting S. D. and Millican P. F. (1998). The role of diet in hatchery conditioning of Pecten maximus L.: A review. *Aquaculture* 165, 167-178. 10.1016/S0044-8486(98)00268-3

[BIO035923C74] Valente L. M. P., Gouveia A., Rema P., Matos J., Gomes E. F. and Pinto I. S. (2006). Evaluation of three seaweeds Gracilaria bursa-pastoris, Ulva rigida and Gracilaria cornea as dietary ingredients in European sea bass (Dicentrarchus labrax) juveniles. *Aquaculture* 252, 85-91. 10.1016/j.aquaculture.2005.11.052

[BIO035923C75] Viles F. J. and Silverman L. (1949). Determination of Starch and Cellulose with Anthrone. *Anal. Chem.* 21, 950-953. 10.1021/ac60032a019

[BIO035923C76] Walne P. R. and Mann R. (1975). Growth and biochemical composition of Ostrea edulis and Crassostrea gigas. In *Proceedings of the 9th European Marine Biology Symposium Oban* (ed. Barnes H.), pp. 587-607. Scotlland, UK: Aberdeen University Press.

[BIO035923C77] Winkler U. K. and Stuckmann M. (1979). Glycogen, hyaluronate, and some other polysaccharides greatly enhance the formation of exolipase by Serratia marcescens. *J. Bacteriol.* 138, 663-670. 222724 10.1128/jb.138.3.663-670.1979 PMC218088

[BIO035923C78] Xuan X., Wen X., Li S., Zhu D. and Li Y. (2013). Potential use of macro-algae Gracilaria lemaneiformis in diets for the black sea bream, Acanthopagrus schlegelii, juvenile. *Aquaculture* 412–413, 167-162 10.1016/j.aquaculture.2013.07.022

