# Supplementary material for: Viability of dietary substitution of live microalgae with dry Ulva rigida in broodstock conditioning of the Pacific oyster (Crassostrea gigas)
Source: Biol Open. 2018 Aug 20;7(9):bio035923. doi: 10.1242/bio.035923 (PMC6176949; doi:10.1242/bio.035923)
Supplement: Supplementary information [file biolopen-7-035923-s1.pdf]

© 2018. Published by The Company of Biologists Ltd | Biology Open (2018): doi:10.1242/bio.035923: Supplementary information

FIRST PERSON

First person – Ana Rato

First Person is a series of interviews with the first authors of a selection of papers published in Biology Open, helping early-career researchers promote themselves alongside their papers. Ana Rato is first author on 'Viability of dietary substitution of live microalgae with dry *Ulva rigida* in broodstock conditioning of the Pacific oyster (*Crassostrea gigas*)', published in BiO. Ana is a research fellow under the supervision of Dr Rodrigo Ozório at CIIMAR and Dr Domitlía Matias at IPMA, Portugal, investigating nutrition, immune and behavioural responses in the aquaculture field.

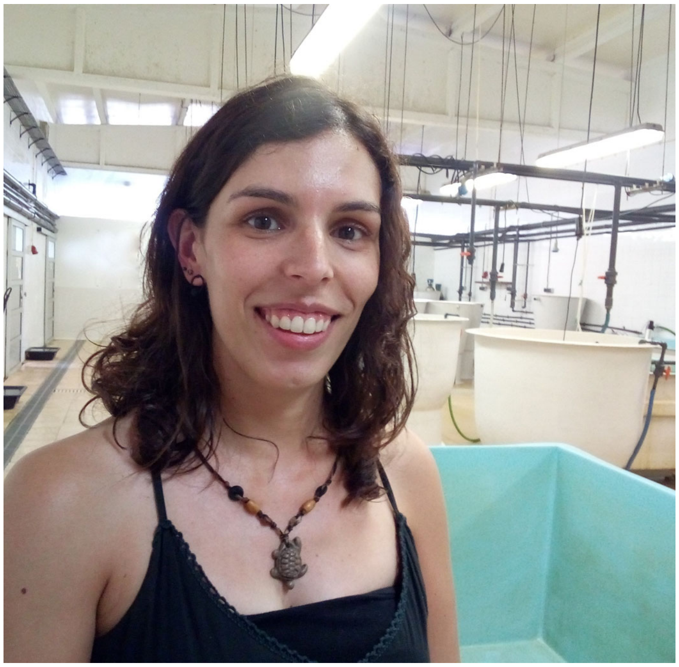

Ana Rato

What is your scientific background and the general focus of your lab?

My scientific background is marine biology. I did my bachelor's and master's degrees at the University of Algarve, Portugal. During my master's thesis I studied endocrine responses in cichlids, namely the role of male pheromones in female endocrine behaviour. Two years ago I embraced a new challenge: the opportunity to work with bivalve aquaculture, mainly oyster aquaculture, which gave rise to this research paper. Both labs, CIIMAR (Interdisciplinary Centre of Marine and Environmental Research) and IPMA (Portuguese Institute for Sea and Atmosphere – Division of Aquaculture and Upgrading), focus on aquaculture and the quality of sea products.

How would you explain the main findings of your paper to non-scientific family and friends?

The diet of bivalves comprises live microalgae, which means that bivalve aquaculture is strongly dependent on live microalgae production. This production represents ∼40% of the operative costs in a bivalve hatchery. Thus, we aimed to find a diet that allowed a reduction in these costs. Pacific oysters were chosen since they are a sea product with extremely high market value and interest worldwide. The results showed that a diet composed of 25% of dry macrolagae (*Ulva rigida*) and 75% of live microalgae may lead to similar reproductive success, nutritional quality and physiological condition to broodstock oysters fed with 100% live microalgae. Our findings suggest that it is possible to reduce operative costs of live microalgae production by 25%, without compromising the quality of oysters.

What are the potential implications of these results for your field of research?

Although there is still so much to know about the effect of macroalgae substitution on the diet of bivalves, this study represents a step towards the future of bivalve feeding. If you could apply this diet in commercial hatcheries, it would represent a reduction in operative costs and reduce dependence on live diets. Scientifically, it means that oysters can ingest 150 µm particles, which opens new lines of research and a whole new world to discover regarding not only bivalves themselves, but also the effects and benefits of macroalgae dietary inclusion.

Ana Rato's contact details: CIIMAR, University of Porto, 4050-208 Matosinhos, Portugal and IPMA, 8700-305 Olhão, Portugal.
E-mail: acrato@outlook.pt

"When I finished the research, I actually had the answers to these questions; however, I have a series of new unanswered questions."

What has surprised you the most while conducting your research?

Firstly, it was amazing to see first-hand how survival and viability are the main 'concern' of a species, even when deprived of food or faced with an inadequate diet. Secondly, when I started this research, one of the goals, besides trying to include macroalgae in the diet of oysters, was to determine if these bivalves were actually able to ingest particles of that size. When I finished the research, I actually had the answers to these questions; however, I have a series of new unanswered questions. The work of science is never finished.

What, in your opinion, are some of the greatest achievements in your field and how has this influenced your research?

Although some alternative bivalve feeding products have emerged in the last decades, good quality inert diets still do not exist. In general, aquaculture research has been focused on the pursuit of alternative products, such as 'waste products', as a way to create alternative diets that minimize the impact of aquaculture and its associated products at ecosystem level, without compromising animal welfare. Some previous research in aquaculture studied the possible introduction of an alternative diet in juvenile bivalves. This research, along with the need for a feasible alternative diet, gave rise to our study. Additionally, following on from the work in this article, it was possible to perform several other trials that allowed us

Biology Open (2018): doi:10.1242/bio.035923: Supplementary information

to evaluate how the nutritional composition of diets influences the functional responses of the organisms, such as oxidative stress and oxygen consumption.

What changes do you think could improve the professional lives of early-career scientists?

Early-career scientists face several problems, the most worrying being limited or non-existent research funding, short-term contracts or research grants, and unstable research positions. Many early-career scientists begin working as volunteers or in short-term research grants, one after another, putting their personal lives on hold in order to pursue the 'dream career'. I believe there is a need to create more funding opportunities, with stable and recognized research positions as well as an improvement in research contracts. This way, early-career scientists would not be forced to choose between professional or personal lives, or leave academia so they can achieve professional stability.

What's next for you?

Science, marine biology and aquaculture research still fascinate me like they did when I was five years old. Despite all problems that research faces, I would like to continue 'making science'. The first step is to do a PhD, and I would also like to try to answer the new questions that arise from this research.

Reference

Rato, A., Joaquim, S., Tavares, T. G., Martins, Z. E., Guedes, A. C., Pereira, L. F., Machado, J., Matias, A. M., Gonçalves, J. F. M., Vaz-Pires, P., Magnoni, L. J., Ozório, R. O. A. and Matias, D. (2018). Viability of dietary substitution of live microalgae with dry *Ulva rigida* in broodstock conditioning of the Pacific oyster (*Crassostrea gigas*). *Biol. Open* **7**: bio035923,
